# Grasping the Intentions of Others with One's Own Mirror Neuron System

**DOI:** 10.1371/journal.pbio.0030079

**Published:** 2005-02-22

**Authors:** Marco Iacoboni, Istvan Molnar-Szakacs, Vittorio Gallese, Giovanni Buccino, John C Mazziotta, Giacomo Rizzolatti

**Affiliations:** **1**Ahmanson-Lovelace Brain Mapping Center, Neuropsychiatric InstituteDavid Geffen School of Medicine, University of California, Los Angeles, CaliforniaUnited States of America; **2**Department of Psychiatry and Biobehavioral Sciences, David Geffen School of MedicineUniversity of California, Los Angeles, CaliforniaUnited States of America; **3**Brain Research Institute, David Geffen School of MedicineUniversity of California, Los Angeles, CaliforniaUnited States of America; **4**Center for Culture, Brainand Development, University of California, Los Angeles, CaliforniaUnited States of America; **5**Department of NeurosciencesUniversity of ParmaItaly; **6**Department of Neurology, David Geffen School of MedicineUniversity of California, Los Angeles, CaliforniaUnited States of America; **7**Department of Pharmacology, David Geffen School of MedicineUniversity of California, Los Angeles, CaliforniaUnited States of America; **8**Department of Radiological Sciences, David Geffen School of MedicineUniversity of California, Los Angeles, CaliforniaUnited States of America; University of MinnesotaUnited States of America

## Abstract

Understanding the intentions of others while watching their actions is a fundamental building block of social behavior. The neural and functional mechanisms underlying this ability are still poorly understood. To investigate these mechanisms we used functional magnetic resonance imaging. Twenty-three subjects watched three kinds of stimuli: grasping hand actions without a context, context only (scenes containing objects), and grasping hand actions performed in two different contexts. In the latter condition the context suggested the intention associated with the grasping action (either drinking or cleaning). Actions embedded in contexts, compared with the other two conditions, yielded a significant signal increase in the posterior part of the inferior frontal gyrus and the adjacent sector of the ventral premotor cortex where hand actions are represented. Thus, premotor mirror neuron areas—areas active during the execution and the observation of an action—previously thought to be involved only in action recognition are actually also involved in understanding the intentions of others. To ascribe an intention is to infer a forthcoming new goal, and this is an operation that the motor system does automatically.

## Abstract 2

Functional magnetic resonance imaging is used to explore the responses of premotor cortical areas to observing the actions of others

## Introduction

The ability to understand the intentions associated with the actions of others is a fundamental component of social behavior, and its deficit is typically associated with socially isolating mental diseases such as autism [1,2]. The neural mechanisms underlying this ability are poorly understood. Recently, the discovery of a special class of neurons in the primate premotor cortex has provided some clues with respect to such mechanisms. Mirror neurons are premotor neurons that fire when the monkey performs object-directed actions such as grasping, tearing, manipulating, holding, but also when the animal observes somebody else, either a conspecific or a human experimenter, performing the same class of actions [3,4,5]. In fact, even the sound of an action in the dark activates these neurons [6,7]. In the macaque, two major areas containing mirror neurons have been identified so far, area F5 in the inferior frontal cortex and area PF/PFG in the inferior parietal cortex [8]. Inferior frontal and posterior parietal human areas with mirror properties have also been described with different techniques in several labs [9,10,11,12,13,14,15,16,17,18,19,20,21].

It was proposed early on that mirror neurons may provide a neural mechanism for understanding the intentions of other people [22]. The basic properties of mirror neurons, however, could be interpreted more parsimoniously, such as that mirror neurons provide a mechanism for recognizing the observed motor acts (e.g., grasping, holding, bringing to the mouth). The mirror neuron mechanism is, in fact, reminiscent of categorical perception [23,24]. For example, some mirror neurons do not discriminate between stimuli of the same category (i.e., the sight of different kinds of grasping actions can activate the same neuron), but discriminate well between actions belonging to different categories, even when the observed actions share several visual features. These properties seem to indicate an action recognition mechanism (“that's a grasp”) rather than an intention-coding mechanism.

Action recognition, however, has a special status with respect to recognition, for instance, of objects or sounds. Action implies a goal and an agent. Consequently, action recognition implies the recognition of a goal, and, from another perspective, the understanding of the agent's intentions. John sees Mary grasping an apple. By seeing her hand moving toward the apple, he recognizes what she is doing (“that's a grasp”), but also that she *wants* to grasp the apple, that is, her immediate, stimulus-linked “intention,” or goal.

More complex and interesting, however, is the problem of whether the mirror neuron system also plays a role in coding the global intention of the actor performing a given motor act. Mary is grasping an apple. Why is she grasping it? Does she want to eat it, or give it to her brother, or maybe throw it away? The aim of the present study is to investigate the neural basis of intention understanding in this sense and, more specifically, the role played by the human mirror neuron system in this type of intention understanding. The term “intention” will be always used in this specific sense, to indicate the “why” of an action.

An important clue for clarifying the intentions behind the actions of others is given by the context in which these actions are performed. The same action done in two different contexts acquires different meanings and may reflect two different intentions. Thus, what we aimed to investigate was whether the observation of the same grasping action, either embedded in contexts that cued the intention associated with the action or in the absence of a context cueing the observer, elicited the same or differential activity in mirror neuron areas for grasping in the human brain. If the mirror neuron system simply codes the type of observed action and its immediate goal, then the activity in mirror neuron areas should not be influenced by the presence or the absence of context. If, in contrast, the mirror neuron system codes the global intention associated with the observed action, then the presence of a context that cues the observer should modulate activity in mirror neuron areas. To test these competing hypotheses, we studied normal volunteers using functional magnetic resonance imaging, which allows in vivo monitoring of brain activity. We found that observing grasping actions embedded in contexts yielded greater activity in mirror neuron areas in the inferior frontal cortex than observing grasping actions in the absence of contexts or while observing contexts only. This suggests that the human mirror neuron system does not simply provide an action recognition mechanism, but also constitutes a neural system for coding the intentions of others.

## Results

**Figure 1:**
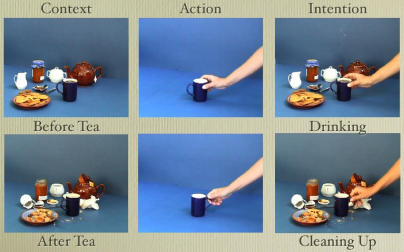
Six Images Taken from the Context, Action, and Intention Clips The images are organized in three columns and two rows. Each column corresponds to one of the experimental conditions. From left to right: Context, Action, and Intention. In the Context condition there were two types of clips, a “before tea” context (upper row) and an “after tea” context (lower row). In the Action condition two types of grips were displayed an equal number of times, a whole-hand prehension (upper row) and a precision grip (lower row). In the Intention condition there were two types of contexts surrounding a grasping action. The “before tea” context suggested the intention of drinking (upper row), and the “after tea” context suggested the intention of cleaning (lower row). Whole-hand prehension (displayed in the upper row of the Intention column) and precision grip (displayed in the lower row of the Intention column) were presented an equal number of times in the “drinking” Intention clip and the “cleaning” Intention clip.

Subjects watched three different types of movie clips (see Figure 1): Context, Action, and Intention, interspersed with periods of blank screen (rest condition). The Context condition consisted of two scenes with three-dimensional objects (a teapot, a mug, cookies, a jar, etc). The objects were arranged either as just before having tea (the “drinking” context) or as just after having tea (the “cleaning” context). The Action condition consisted of a hand grasping a cup in the absence of a context on an objectless background. Two types of grasping actions were shown in the same block an equal number of times: a precision grip (the fingers grasping the cup handle) and a whole-hand prehension (the hand grasping the cup body). In the Intention condition, the grasping actions (also precision grip and whole-hand prehension shown for an equal number of times) were embedded in the two scenes used in the Context condition, the “drinking” context and the “cleaning” context (Figure 1). Here, the context cued the intention behind the action. The “drinking” context suggested that the hand was grasping the cup to drink. The “cleaning” context suggested that the hand was grasping the cup to clean up. Thus, the Intention condition contained information that allowed the understanding of intention, whereas the Action and Context conditions did not (i.e., the Action condition was ambiguous, and the Context condition did not contain any action).

**Figure 2:**
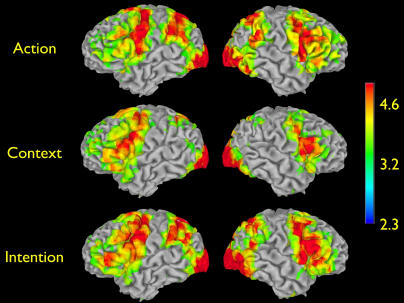
Areas of Increased Signal for the Three Experimental Conditions Threshold of *Z* = 2.3 at voxel level and a cluster level corrected for the whole brain at *p* < 0.05.

Figure 2 displays the brain areas showing significant signal increase, indexing increased neural activity, for Action, Context, and Intention, compared to rest. As expected, given the complexity of the stimuli, large increases in neural activity were observed in occipital, posterior temporal, parietal, and frontal areas (especially robust in the premotor cortex) for observation of the Action and Intention conditions.

Notably, the observation of the Intention and of the Action clips compared to rest yielded significant signal increase in the parieto-frontal cortical circuit for grasping. This circuit is known to be active during the observation, imitation, and execution of finger movements (“mirror neuron system”) [10,11,12,13,14,15,16,17,18,19,20,21,25,26,27]. The observation of the Context clip compared to rest yielded signal increases in largely similar cortical areas, with the notable exceptions of the superior temporal sulcus (STS) region and inferior parietal lobule. The STS region is known to respond to biological motion [28,29], and the absence of the grasping action in the Context condition explains the lack of increased signal in the STS. The lack of increased signal in the inferior parietal lobule is also explained by the absence of an action in the Context condition. Note that, in monkeys, inferior parietal area PF/PFG contains mirror neurons for grasping [8]. Thus, it is likely that the human homologue of PF/PFG is activated by the sight of the grasping action in the Action and Intention conditions, but not in the Context condition, where the action is not presented. The Context condition activates the inferior frontal areas for grasping, even though only graspable objects—but no grasping actions—are shown. In the monkey brain, ventral premotor area F5 contains, in addition to mirror neurons, a population of cells called canonical neurons [4]. These neurons fire during the execution of grasping actions as well as during the passive observation of graspable objects, but not during the observation of an action directed at the graspable object. Neurons with these properties mediate the visuo-motor transformations required by object-directed actions [30,31] and are likely activated by the sight of the Context clips.

**Figure 3:**
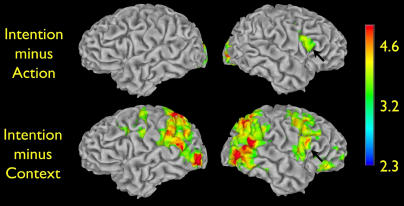
Signal Increases for Intention minus Action and Intention minus Context Threshold of *Z* = 2.3 at voxel level and a cluster level corrected for the whole brain at *p* < 0.05. The black arrow indicates the only area showing signal increase in both comparisons. The area is located in the dorsal sector of pars opercularis, where mirror activity has been repeatedly observed [10,11,12,13,14,15,16,17,18,19,20,27]. See Tables S1 and S2 for coordinates of local maxima.

The critical question for this study was whether there are significant differences between the Intention condition and the Action and Context conditions in areas known to have mirror properties in the human brain. Figure 3 displays these differences. The Intention condition yielded significant signal increases—compared to the Action condition—in visual areas and in the right inferior frontal cortex, in the dorsal part of the pars opercularis of the inferior frontal gyrus (Figure 3, upper row). The increased activity in visual areas is expected, given the presence of objects in the Intention condition, but not in the Action condition. The increased right inferior frontal activity is located in a frontal area known to have mirror neuron properties, thus suggesting that this cortical area does not simply provide an action recognition mechanism (“that's a grasp”) but rather it is critical for understanding the intentions behind others' actions.

To further test the functional properties of the signal increase in inferior frontal cortex, we looked at signal changes in the Intention condition minus the Context condition (Figure 3, lower row). These signal increases were most likely due to grasping neurons located in the inferior parietal lobule, to neurons responding to biological motion in the posterior part of the STS region, and to motion-responsive neurons present in the MT/V5 complex. Most importantly, signal increase was also found in right frontal areas, including the same voxels—as confirmed by masking procedures—in inferior frontal cortex previously seen activated in the comparison of the Intention condition versus Action condition. Thus, the differential activation in inferior frontal cortex observed in the Intention condition versus Action condition, cannot be simply due to the presence of objects in the Intention clips, given that the Context clips also contain objects.

From the contrasts Intention–Action and Intention–Context it is clear that the strongest activity in right inferior frontal cortex is present in the Intention condition. This could be due to two factors, not mutually exclusive: (1) a summation of canonical and mirror neurons activity, and (2) additional activation of mirror neurons of the inferior frontal cortex that code the action the agent will most likely make next. Because in the Intention clips the same action was shown in two contexts (“drinking” and “cleaning”), one can test the intention-coding hypothesis by analyzing the signal increase during observation of the Intention clips. A differential signal increase for the “drinking” Intention clip compared to the “cleaning” Intention clip would indicate neural activity specifically coding the intention of the agent. This logic would hold only if there is no differential signal increase in the “drinking” and “cleaning” Context conditions, when no action is displayed.

**Figure 4:**
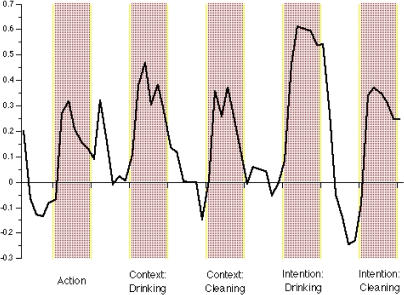
Time Series of the Inferior Frontal Area Showing Increased Signal in the Comparisons Intention minus Action and Intention minus Context The drinking Intention condition yielded a much stronger response than the cleaning Intention condition (*p* < 0.003), whereas no reliable difference was observed between the drinking and cleaning Context conditions (*p* > 0.19). The time series represents the average activity for all subjects in all voxels reaching statistical threshold in the right inferior frontal cortex.

To test this hypothesis, we compared the signal change in the inferior frontal area in the two Intention clips and the two Context clips. The “drinking” Intention clip yielded a much stronger response than the “cleaning” Intention clip (*p* < 0.003; Figure 4). In contrast, no reliable difference was observed between the “drinking” Context clip and the “cleaning” Context clip (*p* > 0.19). These findings clearly show that coding intention activates a specific set of inferior frontal cortex neurons and that this activation cannot be attributed either to the grasping action (identical in both “drinking” and “cleaning” Intention clips) or to the surrounding objects, given that these objects produced identical signal increase in the “drinking” and “cleaning” Context clips, when no action was displayed.

### Automaticity of the Human Mirror Neuron System

We also tested whether a top-down modulation of cognitive strategy may affect the neural systems critical to intention understanding. The 23 volunteers recruited for the experiment received two different kinds of instructions. Eleven participants were told to simply watch the movie clips (Implicit task). Twelve participants were told to attend to the displayed objects while watching the Context clips and to attend to the type of grip while watching the Action clips. These participants were also told to infer the intention of the grasping action according to the context in which the action occurred in the Intention clips (Explicit task). After the imaging experiment, participants were debriefed. All participants had clearly attended to the stimuli and could answer appropriately to questions regarding the movie clips. In particular, all participants associated the intention of drinking to the grasping action in the “during tea” Intention clip, and the intention of cleaning up to the grasping action in the “after tea” Intention clip, regardless of the type of instruction received.

**Figure 5:**
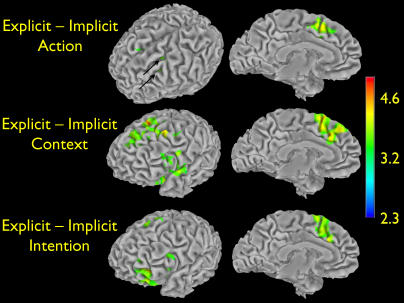
Significant Signal Changes in Subjects Receiving Explicit Instructions Compared to Subjects Receiving Implicit Instructions in the Three Tasks Versus Rest Threshold of *Z* = 2.3 at voxel level and a cluster level corrected for the whole brain at *p* < 0.05. The two black arrows indicate two foci of activity in dorsal premotor cortex that are located deep in the sulci and thus not easily visible on the three-dimensional surface rendering. See Tables S3–S5 for coordinates of local maxima.

The two groups of participants that received the two types of instructions had similar patterns of increased signal versus rest for Action, Context, and Intention (see Figures S1 and S2). The effect of task instructions is displayed in Figure 5. In all conditions, participants that received the Explicit instructions had signal increases in the left frontal lobe, and, in particular, in the mesial frontal and cingulate areas. This signal increase is likely due to the greater effort required by the Explicit instructions [32,33], rather than to understanding the intentions behind the observed actions. In fact, participants receiving either type of instructions understood the intentions associated with the grasping action equally well. Critically, the right inferior frontal cortex—the grasping mirror neuron area that showed increased signal for Intention compared to Action and Context—showed no differences between participants receiving Explicit instructions and those receiving Implicit instructions. This suggests that top-down influences are unlikely to modulate the activity of mirror neuron areas. This lack of top-down influences is a feature typical of automatic processing.

## Discussion

The data of the present study suggest that the role of the mirror neuron system in coding actions is more complex than previously shown and extends from action recognition to the coding of intentions. Experiments in monkeys demonstrated that frontal and parietal mirror neurons code the “what” of the observed action (e.g., “the hand grasps the cup”) [4,6,8,34]. They did not address, however, the issue of whether these neurons, or a subset of them, also code the “why” of an action (e.g., “the hand grasps the cup *in order to drink*”).

The findings of the present study showing increased activity of the right inferior frontal cortex for the Intention condition strongly suggest that this mirror neuron area actively participates in understanding the intentions behind the observed actions. If this area were only involved in action understanding (the “what” of an action), a similar response should have been observed in the inferior frontal cortex while observing grasping actions, regardless of whether a context surrounding the observed grasping action was present or not.

Before accepting this conclusion, however, there are some points that must be clarified. First, one might argue that the signal increase observed in the inferior frontal cortex was simply due to detecting an action in *any* context. That is, it is the complexity of observing an action embedded in a scene, and not the coding of the intention behind actions, that determined the signal increase. A second issue, closely related to the first one, is the issue of canonical neurons. These neurons fire at the sight of graspable objects. Because they are also located in the inferior frontal cortex, one might be led to conclude that the increased activity we observed in the Intention clips was due to the presence of objects. Note, however, that canonical neurons do not fire at the sight of an action directed to a graspable object, even though the object is visible [35].

A strong argument against both these objections is that the activity in inferior frontal cortex is reliably different between “drinking” Intention clips and “cleaning” Intention clips, even though graspable objects were present in both conditions. In contrast, no differences in activity in the inferior frontal region were observed when “drinking” and “cleaning” clips of the Context condition were compared. Thus, the simple presence of an action embedded in a scene is not sufficient to explain the findings. Similarly, the sum of canonical and mirror neurons cannot account for the observed signal increase in the Intention condition, because this increase should be identical for both “drinking” and “cleaning.” Because “drinking” and “cleaning” contexts determined different activations in the Intention condition, it appears that there are sets of neurons in human inferior frontal cortex that specifically code the “why” of the action and respond differently to different intentions.

An important issue to consider in interpreting these data is the relationship between the present results and the activity of single neurons in the activated area. On the basis of our current knowledge of physiological properties of the inferior frontal cortex, the most parsimonious explanation of the findings reported here is that mirror neurons are the likely neurons driving the signal changes in our study. This proposal needs, however, a clarification.

The characteristic property of most mirror neurons is the congruence between their visual and motor properties. A neuron discharging during the execution of grasping also fires during observation of grasping done by another individual. This property cannot account for the present findings, specifically, the differences in response observed between the drinking and cleaning Intention clips. Our results suggest that a subset of mirror neurons in the inferior frontal cortex discharge in response to the motor acts that are most likely to follow the observed one. In other words, in the Intention condition, there is activation of classical mirror neurons, plus activation of another set of neurons coding other potential actions sequentially related to the observed one.

This interpretation of our findings implies that, in addition to the classically described mirror neurons that fire during the execution and observation of the same motor act (e.g., observed and executed grasping), there are neurons that are visually triggered by a given motor act (e.g., grasping observation), but discharge during the execution not of the same motor act, but of another act, functionally related to the observed act (e.g., bringing to the mouth). Neurons of this type have indeed been previously reported in F5 and referred to as “logically related” neurons [34]. In that previous study, however, the role of these “logically related” mirror neurons was never theoretically discussed and their functions remained unclear. The present findings not only allow one to attribute a functional role to these “logically related” mirror neurons, but also suggest that they may be part of a chain of neurons coding the intentions of other people's actions.

What are the possible factors that selectively trigger these “logically related” mirror neurons? The most straightforward interpretation of our results is that the selection of these neurons is due to the observation of an action, also coded by classical mirror neurons, in a context in which that action is typically followed by a subsequent specific motor act. In other words, observing an action carried out in a specific context recalls the chain of motor acts that typically is carried out in that context to actively achieve a goal.

Another possible explanation of how mirror neurons are triggered can be related not only to the context, but also to the way in which the action is performed. It is more common to grasp the handle of the cup with a precision grip while drinking, and to use a whole-hand prehension while cleaning up. Thus, the grasp itself may convey information about the intention behind the grasping action. Although this consideration is very plausible, in general, there are reasons to believe that it is unlikely that this mechanism played a role in our study. First, in all presented grasping actions, when the handle was on the same side of the approaching hand, the grasp was always a precision grip, but when the handle was on the opposite side of the approaching hand, the grasp was always a whole-hand prehension. Thus, the hand always adopted the type of grasp afforded by the orientation of the cup, minimizing the impression that the type of grip would reflect the intentional state of the agent. Second, this hypothesis cannot explain the empirical data. In fact, in both drinking and cleaning Intention clips there was always the same number of precision grips and whole-hand prehensions. However, as Figure 4 shows, the drinking Intention entailed a much larger signal increase than the cleaning Intention. Thus, the differential brain responses in the two Intention clips cannot be explained by a possible meaning conveyed by the grasp type, and cannot even be explained by a possible “compatibility effect” between grasp type and context type (for instance, a whole-hand prehension in a context suggesting cleaning).

The stronger activation of the inferior frontal cortex in the “drinking” as compared to the “cleaning” Intention condition is consistent with our interpretation that a specific chain of neurons coding a probable sequence of motor acts underlies the coding of intention. There is no doubt that, of these two actions, drinking is not only more common and practiced, but also belongs to a more basic motor repertoire, while cleaning is culturally acquired. It is not surprising, therefore, that the chain of neurons coding the intention of drinking is more easily recruited and more widely represented in the inferior frontal cortex than the chain of neurons coding the intention of cleaning.

The conventional view on intention understanding is that the description of an action and the interpretation of the reason why that action is executed rely on largely different mechanisms. In contrast, the present data show that the intentions behind the actions of others can be recognized by the motor system using a mirror mechanism. Mirror neurons are thought to recognize the actions of others, by matching the observed action onto its motor counterpart coded by the same neurons. The present findings strongly suggest that coding the intention associated with the actions of others is based on the activation of a neuronal chain formed by mirror neurons coding the observed motor act and by “logically related” mirror neurons coding the motor acts that are most likely to follow the observed one, in a given context. To ascribe an intention is to infer a forthcoming new goal, and this is an operation that the motor system does automatically.

## Materials and Methods

### 

#### Participants

Through newspaper advertisements we recruited 23 right-handed participants, with a mean age of 26.3 ± 6.3. Eleven participants (six females) received Implicit instructions while 12 participants (nine females) received Explicit instructions. Participants gave informed consent following the guidelines of the UCLA Institutional Review Board. Handedness was determined by a questionnaire adapted from the Edinburgh Handedness Inventory [36]. All participants were screened to rule out medication use, a history of neurological or psychiatric disorders, head trauma, substance abuse, and other serious medical conditions.

#### Image acquisition

Images were acquired using a GE 3.0T MRI scanner with an upgrade for echo-planar imaging (EPI) (Advanced NMR Systems, Woburn, Massachusetts, United States). A two-dimensional spin-echo image (TR = 4,000 ms, TE = 40 ms, 256 by 256, 4-mm thick, 1-mm spacing) was acquired in the sagittal plane to allow prescription of the slices to be obtained in the remaining sequences. This sequence also ensured the absence of structural abnormalities in the brain of the enrolled participants. For each participant, a high-resolution structural T2-weighted EPI volume (spin-echo, TR = 4,000 ms, TE 54 ms, 128 by 128, 26 slices, 4-mm thick, 1-mm spacing) was acquired coplanar with the functional scans. Four functional EPI scans (gradient-echo, TR = 4,000 ms, TE = 25 ms, flip angle = 90, 64 by 64, 26 slices, 4-mm thick, 1-mm spacing) were acquired, each for a duration of 4 min and 36 s. Each functional scan covered the whole brain and was composed of 69 brain volumes. The first three volumes were not included in the analyses owing to expected initial signal instability in the functional scans. The remaining 66 volumes corresponded to six 24-s rest periods (blank screen) and five 24-s task periods (video clips). In each scan there were two Context clips (during tea; after tea), one Action clip, and two Intention clips (drinking; cleaning) (see next section). The order of presentation of the clips was counterbalanced across scans and participants.

#### Stimuli and instructions

There were three different types of 24-s video clips (Context, Action, and Intention). There were two types of Context video clips. They both showed a scene with a series of three-dimensional objects (a teapot, a mug, cookies, a jar, etc). The objects were displayed either as just before having tea (“drinking” context) or as just after having had tea (“cleaning” context). In the Action video clip, a hand was shown grasping a cup in absence of a context on an objectless background. The grasping action was either a precision grip (the hand grasping the cup handle) or a whole-hand prehension (the hand grasping the cup body). The two grips were intermixed in the Action clip. There were two types of Intention video clips. They presented the grasping action in the two Context conditions, the “drinking” and the “cleaning” contexts. Precision grip and whole-hand prehension were intermixed in both “drinking” and “cleaning” Intention clips. A total of eight grasping actions were shown during each Action clip and each Intention clip.

The participants receiving Implicit instructions were simply instructed to watch the clips. The participants receiving Explicit instructions were told to pay attention to the various objects displayed in the Context clips, to pay attention to the type of grip in the Action clip, and to try to figure out the intention motivating the grasping action in the Context clips. All participants were debriefed after the imaging session.

#### Data processing

GE image files were converted in Analyze files and processed with FSL (http://www.fmrib.ox.ac.uk/fsl). Brain volumes within each fMRI run were motion corrected with Motion Correction using the Oxford Centre for Functional Magnetic Resonance Imaging of the Brain (FMRIB) Linear Image Registration Tool (MCFLIRT) [37]. Spatial smoothing was applied using a Gaussian-weighted kernel of 5 mm at full-width half-maximum, and data were high-pass filtered with sigma = 15.0 s and intensity normalized. Functional images were first registered to the co-planar high-resolution structural T2-weighted EPI volume after non-brain structures had been removed with FMRIB's Brain Extraction Tool (BET) from the co-planar high-resolution T2-weighted EPI volume [38]. The co-planar high-resolution structural T2-weighted EPI volume was subsequently registered to the Montreal Neurological Institute Talairach-compatible MR atlas averaging 152 normal subjects using FMRIB's Linear Image Registration Tool (FLIRT) [37].

#### Statistical analyses

Data analyses were performed by modeling the three conditions (Context, Action, and Intention) as stimulus functions, applying the general linear model as implemented in FSL (http://www.fmrib.ox.ac.uk/fsl). Statistical analyses were carried out at three levels: an individual-run level; a higher-order, multiple-runs individual-subject level; and a further higher-order intra- and inter-group comparison level. Time-series statistical analyses were carried out using FMRIB's Improved Linear model (FILM) with local autocorrelation correction [39]. Higher-level intra- and inter-group statistics were carried out using mixed effect (random effects) implemented in FLAME (FMRIB's Local Analysis of Mixed Effects) [40]. *Z* image statistics were performed with a threshold of *Z* = 2.3 at voxel level and a cluster level corrected for the whole brain at *p* < 0.05 [41,42]. The signal change displayed in Figure 4 was statistically analyzed with repeated measures ANOVA and subsequent planned contrasts.

## Supporting Information

Figure S1 Significant Signal Changes in Subjects Receiving Implicit Instructions for Each Task Versus Rest With a threshold of *Z* = 2.3 at voxel level and a cluster level corrected for the whole brain at *p* < 0.05.
(1 MB JPG).

Figure S2 Significant Signal Changes in Subjects Receiving Explicit Instructions for Each Task Versus Rest With a threshold of *Z* = 2.3 at voxel level and a cluster level corrected for the whole brain at *p* < 0.05.
(1.1 MB JPG).

Table S1 Intention minus Action Local maxima in Talairach coordinates. Only the six local maxima with highest *Z* score in each cluster are provided in the table. Cluster size is in voxels. When the cluster encompasses more than one anatomical location, the localization given corresponds to the local maxima with the highest *Z* score.
(82 KB PDF).

Table S2 Intention minus Context Local maxima in Talairach coordinates. Only the six local maxima with highest *Z* score in each cluster are provided in the table. Cluster size is in voxels. When the cluster encompasses more than one anatomical location, the localization given corresponds to the local maxima with the highest *Z* score. LH, left hemisphere; RH, right hemisphere; TPO, temporo-parieto-occipital.
(82 KB PDF).

Table S3 Effect of Task Instructions: Explicit minus Implicit Instruction, Action Versus Rest Local maxima in Talairach coordinates. Only the six local maxima with highest *Z* score in each cluster are provided in the table. Cluster size is in voxels. When the cluster encompasses more than one anatomical location, the localization given corresponds to the local maxima with the highest *Z* score. SMA, supplementary motor area.
(82 KB PDF).

Table S4 Effect of Task Instructions: Explicit minus Implicit Instruction, Context Versus Rest Local maxima in Talairach coordinates. Only the six local maxima with highest *Z* score in each cluster are provided in the table. Cluster size is in voxels. When the cluster encompasses more than one anatomical location, the localization given corresponds to the local maxima with the highest *Z* score.
(82 KB PDF).

Table S5 Effect of Task Instructions: Explicit minus Implicit Instruction, Intention Versus Rest Local maxima in Talairach coordinates. Only the six local maxima with highest *Z* score in each cluster are provided in the table. Cluster size is in voxels. When the cluster encompasses more than one anatomical location, the localization given corresponds to the local maxima with the highest *Z* score. ACC, anterior cingulate cortex; VLPFC, ventrolateral prefrontal cortex.
(82 KB PDF).

## Abbreviations

EPI: echo-planar imaging
FMRIB: Oxford Centre for Functional Magnetic Resonance Imaging of the Brain
STS: superior temporal sulcus
