# Supplementary material for: Grasping the Intentions of Others with One's Own Mirror Neuron System
Source: PLoS Biol. 2005 Feb 22;3(3):e79. doi: 10.1371/journal.pbio.0030079 (PMC1044835; doi:10.1371/journal.pbio.0030079)
Supplement: Supplementary file 1 [file pbio.0030079.st001.pdf]

Supplementary Table 1**. INTENTION minus ACTION. Local Maxima in Talairach coordinates. Only the six local maxima with highest Z score in each cluster are provided in the Table. Cluster size is in voxels. In all these Tables, when the cluster encompasses more than one anatomical location, the localization given corresponds to the local maxima with the highest Z score.**

Localization	Cluster size	Z	x	y	z
Calcarine/Occipital Cortex	8470	5.76	16	-92	0
		5.54	14	-88	-16
		5.53	10	-82	-18
		5.48	28	-80	-22
		5.32	-10	-88	-14
		5.3	10	-92	0
Medial Parietal/Precuneus	1357	3.89	4	-66	52
		3.86	4	-70	40
		3.86	-2	-58	52
		3.74	2	-56	60
		3.55	-4	-54	42
		3.52	10	-58	50
Inferior Frontal Gyrus	581	3.86	46	20	22
		3.58	44	10	34
		3.51	58	28	14
		3.43	38	20	22
		3.42	56	22	22
		3.4	38	16	24
