# Supplementary material for: Grasping the Intentions of Others with One's Own Mirror Neuron System
Source: PLoS Biol. 2005 Feb 22;3(3):e79. doi: 10.1371/journal.pbio.0030079 (PMC1044835; doi:10.1371/journal.pbio.0030079)
Supplement: Supplementary file 2 [file pbio.0030079.st002.pdf]

Supplementary Table 2**. INTENTION minus CONTEXT. Local Maxima in Talairach coordinates. Only the six local maxima with highest Z score in each cluster are provided in the Table. Cluster size is in voxels. TPO: Temporo-Parieto-Occipital. LH: left hemisphere. RH: right hemisphere.**

Localization	Cluster Size	Z	x	y	z
TPO junction	18053	6.04	-34	-46	48
		5.99	-26	-54	62
		5.79	-50	-66	0
		5.69	54	-68	-8
		5.61	-28	-42	52
		5.61	-54	-80	0
Premotor cortex (RH)	2935	4.85	48	8	44
		4.73	50	18	42
		4.41	38	18	34
		4.4	54	28	20
		4.31	22	-6	62
		4.26	36	20	50
Premotor cortex (LH)	1408	4.53	-28	-2	68
		4.11	-24	-8	72
		4.06	-34	-8	50
		4.04	-38	-6	48
		3.61	-24	-10	54
		3.58	-28	-10	58
OrbitoFrontal Cortex	1095	4.3	34	48	6
		4.03	52	38	-14
		3.94	48	42	-16
		3.88	44	50	-10
		3.84	48	50	-10
		3.79	26	52	6
Cerebellum (LH)	1025	4.51	-28	-50	-40
		4.14	-34	-52	-36
		4.14	-18	-70	-36
		4.06	-18	-76	-50
		4.05	-38	-54	-36
		4.04	-20	-74	-46
Cerebellum (RH)	622	4.24	28	-54	-36
		4.04	36	-44	-38
		3.74	40	-54	-34
		3.55	46	-56	-38
		3.54	30	-44	-60
		3.53	36	-56	-34
