# Supplementary material for: Grasping the Intentions of Others with One's Own Mirror Neuron System
Source: PLoS Biol. 2005 Feb 22;3(3):e79. doi: 10.1371/journal.pbio.0030079 (PMC1044835; doi:10.1371/journal.pbio.0030079)
Supplement: Supplementary file 3 [file pbio.0030079.st003.pdf]

Supplementary Table 3. **Effect of task instructions. Local maxima in Talairach coordinates. Explicit minus Implicit instruction, ACTION versus rest. Only the six local maxima with highest Z score in the only cluster surviving the statistical threshold are provided in the Table. Cluster size is in voxels. SMA: Supplementary motor area.**

Localization	Cluster size	Z	x	y	z
Pre-SMA	576	4.22	-6	14	52
		4.08	-2	16	62
		3.65	-6	18	46
		3.63	-2	20	52
		3.4	-6	24	48
		3.32	-6	4	54
