# Supplementary material for: Grasping the Intentions of Others with One's Own Mirror Neuron System
Source: PLoS Biol. 2005 Feb 22;3(3):e79. doi: 10.1371/journal.pbio.0030079 (PMC1044835; doi:10.1371/journal.pbio.0030079)
Supplement: Supplementary file 4 [file pbio.0030079.st004.pdf]

Supplementary Table 4. **Effect of task instructions. Local maxima in Talairach coordinates. Explicit minus Implicit instruction, CONTEXT versus rest. Only the six local maxima with highest Z score in each cluster are provided in the Table. Cluster size is in voxels.**

Localization	Cluster size	Z	x	y	z
Lateral and mesial premotor cortex	2259	4.68	-12	16	60
		4.29	-10	22	62
		4.27	-6	16	58
		4.24	-34	-2	40
		4.21	-22	22	52
		4.19	-6	16	52
Inferior Frontal Gyrus	1211	4.05	-48	28	-12
		3.79	-44	24	10
		3.5	-58	14	26
		3.41	-48	40	-12
		3.4	-62	8	28
		3.32	-54	26	-2
Ventral Striatum	453	3.85	-18	6	2
		3.83	-16	12	4
		3.6	-24	4	-8
		3.56	-22	-8	-2
		3.38	-30	-14	-16
		3.04	-26	-10	-8
