# Supplementary material for: Grasping the Intentions of Others with One's Own Mirror Neuron System
Source: PLoS Biol. 2005 Feb 22;3(3):e79. doi: 10.1371/journal.pbio.0030079 (PMC1044835; doi:10.1371/journal.pbio.0030079)
Supplement: Supplementary file 5 [file pbio.0030079.st005.pdf]

Supplementary Table 5. **Effect of task instructions. Local maxima in Talairach coordinates. Explicit minus Implicit instruction, INTENTION versus rest. Only the six local maxima with highest Z score in each cluster are provided in the Table. Cluster size is in voxels. ACC: Anterior cingulate cortex. VLPFC: ventrolateral prefrontal cortex.**

Localization	Cluster size	Z	x	y	z
PreSMA/ACC	1002	4.11	-8	26	46
		3.97	-2	14	62
		3.55	14	30	30
		3.55	-4	16	52
		3.51	-8	24	62
		3.42	-6	30	36
VLPFC	882	4.13	-38	46	4
		3.52	-40	30	6
		3.49	-34	52	4
		3.39	-26	50	8
		3.33	-40	52	-6
		3.33	-46	24	24
